# Byron’s bear and dwarf rabbits in the classroom: a review of animals against academic stress

**DOI:** 10.3389/fvets.2025.1693505

**Published:** 2025-12-17

**Authors:** Thomas C. Erren, Florian Glenewinkel, Ursula Wild, Jonas Wallraff, Philip Lewis

**Affiliations:** Institute and Policlinic for Occupational Medicine, Environmental Medicine and Prevention Research, Faculty of Medicine and University Hospital of Cologne, University of Cologne, Cologne, Germany

**Keywords:** support animals, pets, human-animal interactions, school, university, academia, academic stress

## Abstract

**Background:**

Coping strategies for academic stress at different stages of human development are important. We address the hypothesis that human-animal interactions (HAI) in both non-therapy and therapy situations can help adults, adolescents and children cope with academic stress in universities and schools. We point to Lord Byron’s “pet” bear in the 19th century and to media reports of pets on the high-pressure academic campuses of Oxford and Cambridge as historical and current examples.

**Methods:**

We review scientific literature concerning HAI and stress in university settings (exemplified via narrative information) and in the pre-university school environment (explored via systematic information). Media reports from Oxbridge underline the popularity of HAI.

**Results:**

A pilot search returned *n* = 4 recent reviews and meta-analyses of studies from university settings that covered mental, cognitive, and physiological health outcomes. The synthesis of these studies suggests that HAI may have benefits for stress and anxiety with the optimal duration and frequency of HAI remaining open. Studies from pre-university settings also suggest a possible benefit in terms of reducing anxiety and salivary cortisol as an indicator of stress, but there are only a few studies on this topic (*n* = 3).

**Conclusion:**

There is potential for improved mental health outcomes through HAI in academic environments, but more research is needed to establish best practices.

## 1 Introduction

In the early 1800s, Lord Byron was not happy about the rule that forbade students from bringing a dog as a pet to Cambridge (1, 2). Thus, the eccentric poet got himself a “pet” bear (unlike dogs, bears were not expressly forbidden)! How Byron’s bear affected academic stress – for him and for others on campus – is an open question.

Academic stress can affect people in childhood, adolescence, and/or adulthood, in schools and/or in universities. It can be defined as pressure and tension arising from the requirements of performance in school and university settings. It can cause negative effects in performance and well-being, including increased anxiety and depression (3). Positive human-animal interactions (HAIs) can reduce depression and loneliness and promote social contact and physical activity (4).

How HAI in academic settings affect people also depends on their attitudes and beliefs toward animals. Individuals who have a positive attitude toward animals may experience greater stress reduction, engagement, and learning benefits during animal-assisted activities (5). Frameworks like the “One Health-One Welfare” approach emphasize the emotional effects of interspecies interactions (6). We explore the hypothesis that HAI can help children, adolescents, and adults cope with academic stress. In a previous review of the effects of AAA (animal-assisted activities) on stress in healthcare workers, AAA was associated with reduced stress and tiredness, improved mood, and positive perceptions and experiences of the AAA (7).

We address the hypothesis in three parts. We point to interactions with pets on the Oxford and Cambridge University campuses (as examples of high-pressure academic settings) that have been reported in the media (non-systematic gray literature). We consider the scientific literature concerning HAI and stress in university settings. We consider the scientific literature concerning HAI and stress in pre-university school settings. Importantly, while media/gray sources serve as background information only, conclusions are based on peer-reviewed evidence. We close with a discussion of our findings and suggestions.

## 2 Materials and methods

We work with a mixed or dual methodological approach by using narrative information from media reports and recent reviews to exemplify HAI in university settings (narrative information) and explore scientific evidence from primary research in school settings (systematic information).

### 2.1 A note on human-animal interactions

To explore the effect of animals on humans in non-therapeutic and therapeutic situations, we use the umbrella term Human-Animal Interactions (HAI). HAI encompass therapy animals, facility animals, support animals, and visiting animals; some of these animals may provide animal-assisted services (AAS) (8), animal-assisted therapy (AAT), animal-assisted intervention (AAI), animal-assisted activities (AAA), and animal-assisted prevention (AAP) (7). This list is not exhaustive.

### 2.2 Media reports regarding Oxford and Cambridge

Through a non-systematic gray literature search (Google), media sources covering pets on the campuses of Oxford and Cambridge Universities were identified (Table 1).

**Table 1:** Media reports of pets brought to Oxford and Cambridge.

Pets	Headline
Alpacas	• Noise from ‘wellbeing alpacas’ at Oxford University leaves revising students spitting mad (32) • JCR students welcome some four-legged friends to St Hughs! (33)
Cats	• Wags in the rag (34) • In pictures: the cats of Cambridge (35) • Cambridge University’s animals help students de-stress (16)
Dogs	• The campus canine healing stressed Oxford students (36) • De-stress with fluffy friends: Wellbeing Dog Walk (14) • Oxford University students de-stress by cuddling canines (37) • A Walking Interview with Geoffrey Biscuit: Merton’s New Puppy (15) • Why Cambridge needs more therapy animals (38) • Dogs ‘prevent stressed students dropping out’ (39) • Cambridge University’s animals help students de-stress (16)
Goats	• Fitzwilliam just goat a new college pet, and his name is Leo (40)
Guinea pigs	• Cambridge University’s animals help students de-stress (16) • Pet guinea pigs offered places at Lucy Cavendish (41)

### 2.3 Studies at university

A pilot search of the literature concerning HAI and stress in the university setting revealed reviews recently published on this topic. These reviews are narratively synthesized.

### 2.4 Studies at school

#### 2.4.1 Systematic review

A pilot systematic search of the literature concerning HAI and stress in the pre-university setting revealed no systematic review concerning this theme. Thus, we conducted a systematic literature review. Our PICOS definitions, search strategy, eligibility criteria, and data extraction and synthesis plan are described below. This review was conducted following PRISMA principles (9). All screening steps and critical appraisals were conducted independently by at least two authors. Differences were reconciled by the team of authors. The search was conducted on 02 June 2025.

#### 2.4.2 PICOS: population, intervention, comparison, outcome, study design

The population of interest is students in primary or secondary school (i.e., pre-university setting). The intervention or exposure are interactions with animals that are not studies of the animals themselves (e.g., incorporation of animals into tasks, such as reading to an animal, is included, whereas interaction for teaching purposes only, such as anatomy, is excluded). The interaction may have been explicitly observed by researchers or recounted by the participants. Comparators include different levels of interactions (e.g., different animals or no animal, different durations, different settings). Outcomes include physiological or subjective measures of stress or anxiety. These can include validated measures, basic self-reports, or qualitative information. No specific study designs were excluded.

#### 2.4.3 Search strategy

We screened the PubMed and Web of Science databases using relevant search terms combined with Boolean operators (Table 2). Given the specificity of the population of interest, population-based terms included the specification that they can be found in article titles. All other search terms were searched for in titles or abstracts. Specific animal terms were based on those identified from an unsystematic search. Identified studies were imported into the reference manager EndnoteTM (Clarivate Analytics, Philadelphia, U. S.) for further screening. Duplicates and articles not in English or German were removed first. Titles and abstracts were screened against pre-defined eligibility criteria (Table 2). These criteria were based on PICOS definitions. Full text screening and data extraction was done using CovidenceTM systematic review software (Veritas Health Innovation, Melbourne, Australia). Citation searching was done to identify additional papers that fit with our eligibility criteria.

**Table 2:** Search engines, string, and inclusion/exclusion criteria.

Search engines	PubMed, WoS core collection
PubMed search string	(“student*”[ti] OR “class”[ti] OR “classes”[ti] OR “classroom”[ti] OR “school*”[ti] OR “pupil*”[ti]) AND (“dog”[Title/Abstract] OR “dogs”[Title/Abstract] OR “cat”[Title/Abstract] OR “cats”[Title/Abstract] OR “alpaca*”[Title/Abstract] OR “guinea pig*”[Title/Abstract] OR “goat*”[Title/Abstract] OR “pet”[Title/Abstract] OR “pets”[Title/Abstract] OR “animal*”[Title/Abstract] OR “rabbit*”[Title/Abstract] OR “hamster*”[Title/Abstract] OR “horse*”[Title/Abstract] OR “lizard*”[Title/Abstract] OR “snake*”[Title/Abstract] OR “reptile*”[Title/Abstract] OR “fish”[Title/Abstract] OR “frog*”[Title/Abstract] OR “bird*”[Title/Abstract] OR “rodent*”[Title/Abstract]) AND (“stress”[Title/Abstract] OR “anxiety”[Title/Abstract] OR “cortisol”[Title/Abstract] OR “heart rate”[Title/Abstract] OR “blood pressure”[Title/Abstract] OR “HRV”[Title/Abstract])
WoS search string	(TI = (“school*” OR “pupil*” OR “student*” OR “class” OR “classes” OR “classroom”)) AND ((AB = ((“dog” OR “dogs” OR “cat” OR “cats” OR “alpaca*” OR “guinea pig*” OR “goat*” OR “pet” OR “pets” OR “animal*” OR “rabbit*” OR “hamster*” OR “horse*” OR “lizard*” OR “snake*” OR “reptile*” OR “fish” OR “frog*” OR “bird*” OR “rodent*”) AND (“stress” OR “anxiety” OR “cortisol” OR “heart rate” OR “blood pressure” OR “HRV”))) OR (TI = ((“dog” OR “dogs” OR “cat” OR “cats” OR “alpaca*” OR “guinea pig*” OR “goat*” OR “pet” OR “pets” OR “animal*” OR “rabbit*” OR “hamster*” OR “horse*” OR “lizard*” OR “snake*” OR “reptile*” OR “fish” OR “frog*” OR “bird*” OR “rodent*”) AND (“stress” OR “anxiety” OR “cortisol” OR “heart rate” OR “blood pressure” OR “HRV”))) OR ((AB = (“dog” OR “dogs” OR “cat” OR “cats” OR “alpaca*” OR “guinea pig*” OR “goat*” OR “pet” OR “pets” OR “animal*” OR “rabbit*” OR “hamster*” OR “horse*” OR “lizard*” OR “snake*” OR “reptile*” OR “fish” OR “frog*” OR “bird*” OR “rodent*”)) AND (TI = (“stress” OR “anxiety” OR “cortisol” OR “heart rate” OR “blood pressure” OR “HRV”))) OR ((TI = (“dog” OR “dogs” OR “cat” OR “cats” OR “alpaca*” OR “guinea pig*” OR “goat*” OR “pet” OR “pets” OR “animal*” OR “rabbit*” OR “hamster*” OR “horse*” OR “lizard*” OR “snake*” OR “reptile*” OR “fish” OR “frog*” OR “bird*” OR “rodent*”)) AND (AB = (“stress” OR “anxiety” OR “cortisol” OR “heart rate” OR “blood pressure” OR “HRV”))))
Inclusion criteria	(1) Primary research in English or German (2) Population/Context: Students/pupils in primary or secondary school – or equivalent (i.e., not in higher education) (3) Intervention: planned/intended interaction with an animal (4) Outcome: physiological or subjective measures/assessment of stress or anxiety related to studies/school life
Exclusion criteria	(1) Specified curricular-based animal interaction for teaching purposes regarding the animal only (e.g., anatomy).

WoS, Web of Science.

#### 2.4.4 Data extraction and synthesis

PICOS-related data were extracted and tabulated from the pool of relevant articles that remained after the final screening step. Study findings and critical appraisal are presented in a narrative synthesis.

## 3 Results

### 3.1 Media reports regarding Oxford and Cambridge

Given sources of stress galore, it comes as no surprise that leading universities like Oxford and Cambridge (“Oxbridge”) are known for pet interactions on campus (10).

**Figure 1:**
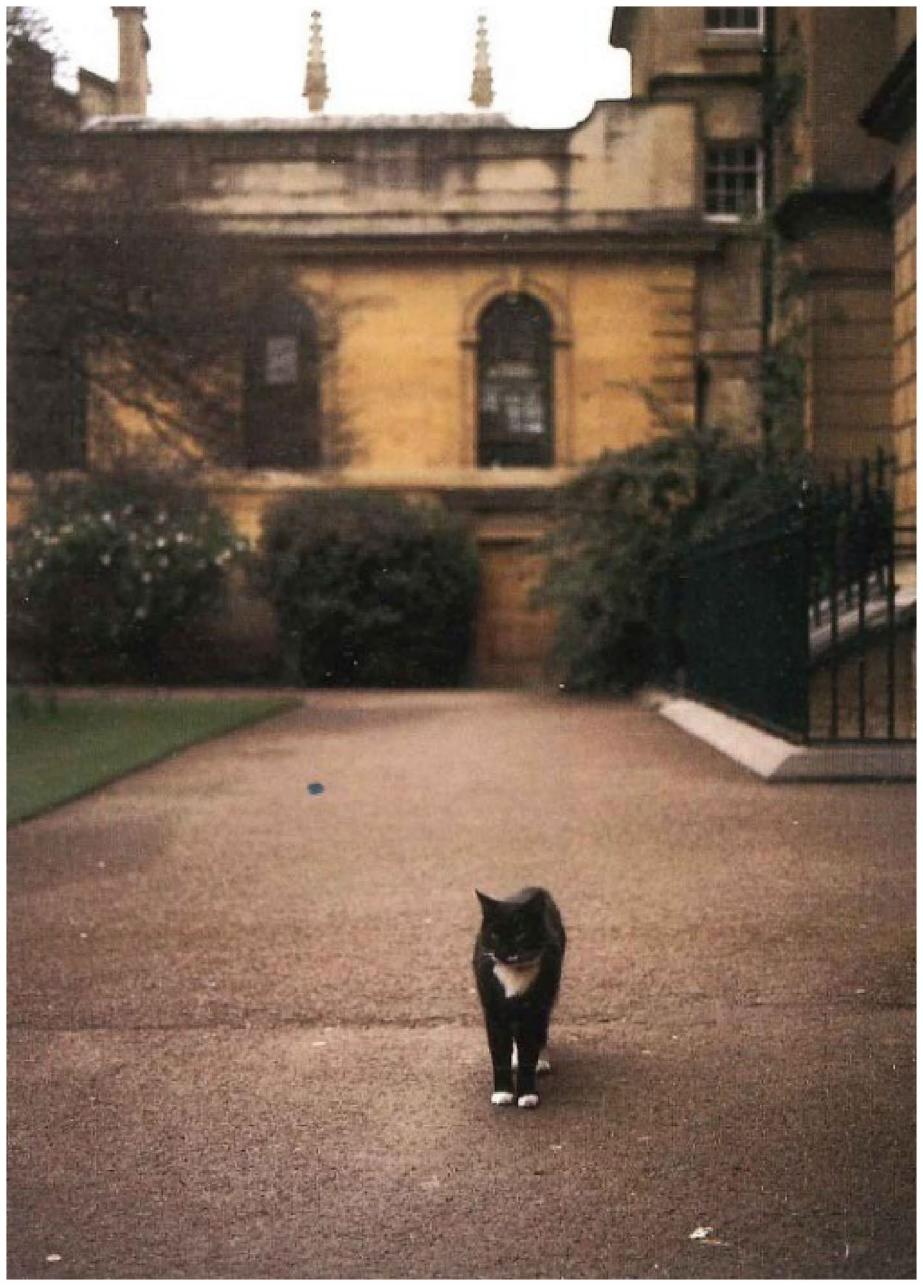
“The Simpkin Dynasty: For many decades, one of Oxford’s most loved and most notorious inhabitants has been Simpkin, the Hertford College cat” (1). Simpkin II (“Cat in residence” 1986–1999). @ https://www.hertford.ox.ac.uk/alumni/hertford-today/the-simpkin-dynasty/simpkins-simpkin-ii/. Reproduced with permission of the Principal, Fellows and Scholars of Hertford College, Oxford. 1. The Simpkin Dynasty (2025). Available online at: https://web.archive.org/web/20250710132338/https://www.hertford.ox.ac.uk/alumni/hertford-today/the-simpkin-dynasty (archived on 10-07-2025) (Accessed July 31, 2025).

The University of Oxford has several pets and other animals on campus, including cats, staff dogs, and wildlife deer and foxes (11, 12). Some colleges have a tortoise and Corpus Christi college hosts an annual tortoise race (11, 13). At some colleges, students can play with dogs, pet them, and take them for walks. As a break from nerve-wracking days, the Oxford Student Union organizes the Wellbeing Dog Walk with outdoor exercise and fresh air (14). Wolfson College’s cocker spaniel Jack helps students to relax and de-stress, as does the golden retriever Geoffrey Biscuit at Merton, and the Simpkin dynasty cats at Hertford College (Figure 1) (15).

The University of Cambridge is also home to pets and pet events for students. For example, students can walk Jack, the spaniel from Corpus Christi, or pet a guinea pig at Lucy Cavendish College (16). The Cambridge University Cat Club has the Facebook intro “Cambridge can be tough. So here are some cute kitties” (17). A very popular event is “Tea with Jasper” – the University’s Marshall Library mascot was once photographed with the Financial Times (16). Students can interact with the cat to help relieve exam stress. Lastly, many animals have been temporarily brought to the campuses of the University of Oxford and the University of Cambridge (Table 1).

### 3.2 Studies at university

In a pilot search, we identified four systematic reviews since 2021 that include HAI for university students; namely, those by Parbery-Clark et al. (18), Huber et al. (19), Manville et al. (20), and Sim et al. (21). The reviews by Huber et al. and Sim et al. include meta-analyses (19, 21). Huber et al. focus on mental, cognitive and physiological health outcomes from RCTs, while Sim et al. focus on RCTs of canine-assisted therapy (with the acronym ‘CAT’!) specifically; thus, involving trained and accredited dogs and their handlers (19, 21). Of the reviews without meta-analyses, Parbery-Clark et al. focus on randomized controlled trials (RCTs) of HAI to improve mental health outcomes, Manville et al. focus on canine-assisted interventions (CAI) (18, 20). Rather than conducting a further systematic review, we synthesize these timely reviews.

Parbery-Clark et al. assessed 11 studies, excluding preexisting psychological diagnoses (e.g., autism, ADHD) and the use of pets or other animals belonging to the study participants themselves (18). All but one study (which used horses) used dogs (various breeds). The specifics of the interventions varied in length (up to 90 min), group size (from individual sessions up to 14 persons per dog), and frequency (seven studies assessed a single session). As outcomes, stress was reported in two studies and anxiety in seven studies, all assessed by questionnaires. Timing of measurements varied widely, taking place immediately after intervention in six studies with four studies adding an experimental stressor before measurement. Only three studies are reported to have done a follow-up longer than 1 week (maximum of 4 months). Regarding results, the authors conclude that the evidence is suggestive for short-term reductions in anxiety with only limited evidence for a reduction in stress.

Manville et al. consider 37 studies with dogs outcomes including “mental health, well-being, stress, anxiety or depression” (20). Sample sizes were varied (*n* = 44–1960) as were dog roles (“friendly,” “companion,” “therapy,” “guide”), dog breeds, and study designs. The specifics of the interventions varied, including by duration (up to 90 min), group size (individual sessions to 14 persons per dog), and frequency (seven studies used a single session). Thirteen of 16 studies identified reduced anxiety. Seventeen of 22 studies identified reduced stress. Questionnaire and physiology techniques were used to assess outcomes.

Huber et al. reviewed 32 studies with mixed results (19). Most physiological metrics (heart rate, blood pressure) show no change post-intervention. Only salivary cortisol is reported as showing small to moderate changes following interventions. Acute anxiety and stress are reported to show a reduction post-intervention in most studies.

Sim et al. reviewed 15 studies and identify reduced stress and anxiety in their meta-analysis of CAT (21). Furthermore, in sub-group analyses of duration, shorter duration interventions (0–14 min) appear to be more consistent in terms of offering benefit; however, these studies have smaller sample sizes and the authors note a potential small-studies effect bias.

Six studies overlap between Manville et al. and Parbery-Clark et al. (18, 20). Of the 15 studies reviewed by Sim et al., only seven overlap with Huber et al. and four with Parbery-Clark et al. (18, 19, 21). Overall, all reviews arrive at the conclusion that the HAI may provide benefit against stress and anxiety in university students, at least with short duration interventions.

The review by Parbery-Clark et al. highlights open questions about continued HAI and the possibility of diminishing returns (i.e., with each return session, the impact of the intervention may diminish) (18). Manville et al. include more extensive material as to why mental health issues are prevalent in this population and also discuss possible social desirability bias in longitudinal studies (20). Huber et al. note the potential scalability of HAI for use at universities and difficulties with other mental health support strategies (19). Sim et al. note CAT may need to be part of a multi-faceted approach to support the mental health of university students (21).

### 3.3 Studies at school

**Figure 2:**
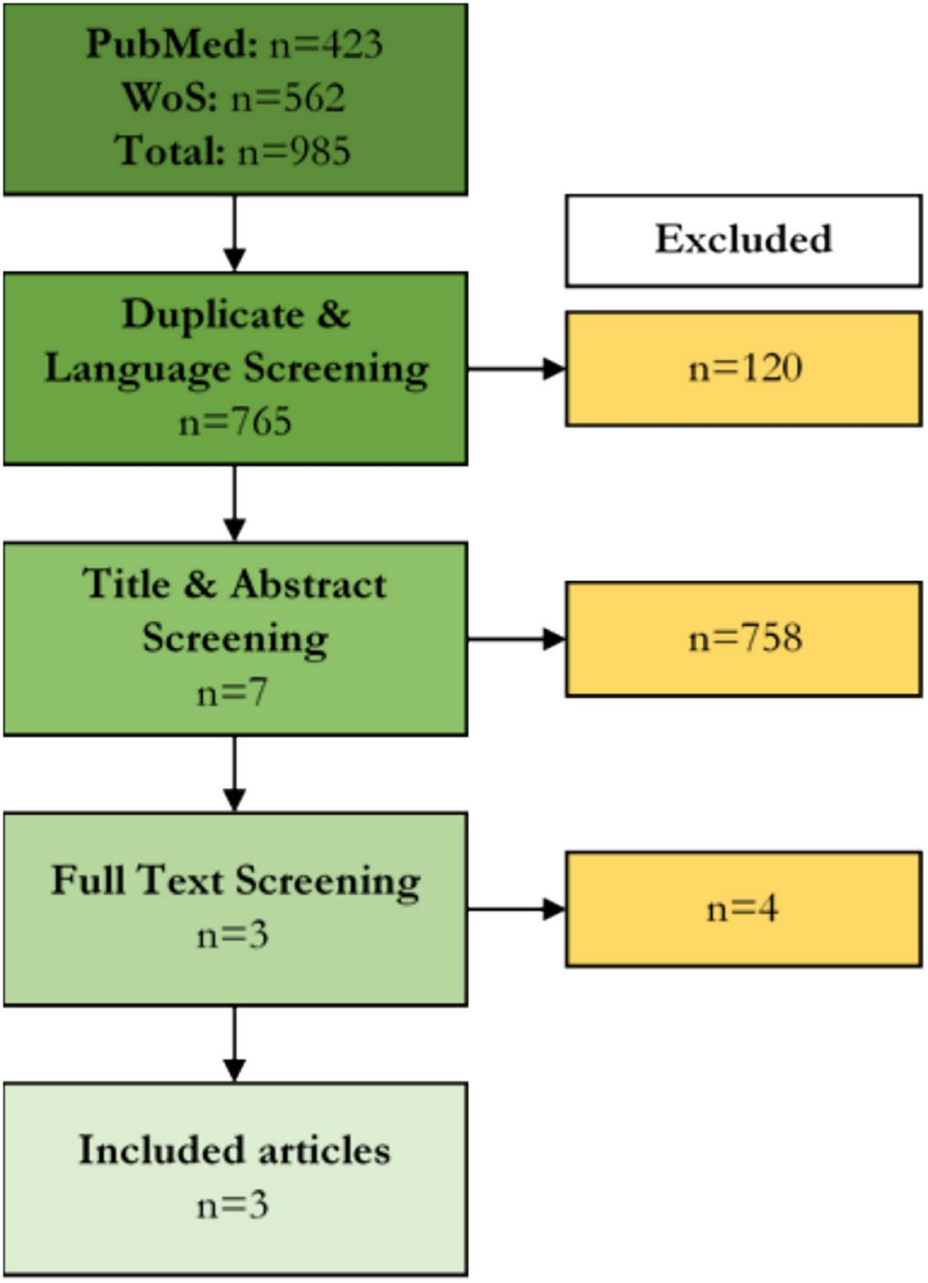
PRISMA flow diagram. WoS, Web of Science.

From the 985 articles returned from database screening, three fit our eligibility criteria. The flow of articles through the screening stages is illustrated in Figure 2.

An overview of the studies is presented in Table 3. The studies investigated the effects of HAI in primary school children.

**Table 3:** Overview of included studies regarding studies at school.

Author (Year)	Population/Data	Exposure/comparator	Outcome	Statistics	Results
Molnár et al. (22)	*N* = 27 first-grade school children (*n* = 19 from mainstream school; *n* = 8 from integrating school), Hungary	6 weeks with rabbit in class (freely moving) and additional pet-time when correctly answering questions vs. 6 weeks without rabbit, repeated cycles	State–Trait Anxiety Inventory for Children (STAI-C); every 3 weeks	Generalized Linear Model	↓Anxiety scores
Iváncsik et al. (23)	*N* = 16 first grade primary school students (age 6–8 years; 50% female), Hungary	6 weeks with rabbit in class (freely moving); weekly AAI sessions with teachings about animals, care, and rabbit interaction vs. 6 weeks without rabbit, repeated cycles	(STAI-C) every 3 weeks; basic skills in children assessed by (every 3 weeks)	Repeated measures ANOVA (with Bonferroni correction)	↓State and trait anxiety (more pronounced in children with high baseline trait anxiety).
Meints et al. (24)	Primary school children from 4 mainstream schools (*n* = 90; age 8.2–10.1 years; *n* = 51 female) and 7 SEN* (*n* = 44; age 8.3–11.4 years; *n* = 6 female), UK	4 weeks of either two sessions of 20 min of group/individual dog-assisted intervention (e.g., greeting, petting, learning about, and interacting with dog) vs. 20 min of relaxation intervention (e.g., finger/toe wriggling, listening to a story, another active part) vs. regular class (control group)	Salivary cortisol at baseline (week 1) and at end of study (week 6) (longer term pre vs. post) & Immediately before and after one intervention in weeks 2, 3, and 5 (acute; pre vs. post; neurotypical participants only)	Repeated measures ANOVA (with Bonferroni correction)	Baseline: ↔ Cortisol between those with/without a dog Longer Term Pre-Post Neurotypical: ↑Cortisol in controls, ↔ in relaxation, ↔ in HAI Longer Term Pre-Post SEN: ↔ Cortisol in controls, ↔ in relaxation, ↓ in HAI (but only for those in group sessions) Acute Pre-Post Neurotypical: ↓Cortisol in all groups

*SEN: special educational needs; ↓ = decreasing; ↑ = increasing; ↔ = no effect.

Two quasi-experimental studies (pretest-posttest designs, no cross-over) (22, 23) assessed the effect of HAI on anxiety in first-grade children starting primary school using the State–Trait Anxiety for Children questionnaire. In alternating six-week intervals (over a total of 24 weeks) a dwarf rabbit (bred for trustworthiness, stress tolerance, and selected according to their boldness and low cortisol levels) was either present or not in a classroom. When present, the animal was free to move and interact with the children. Additionally, once per week, there were sessions wherein the children learned about animals and were enabled to closely interact (e.g., pet) with the rabbit. Children answering questions correctly were given more opportunities to interact with the rabbit.

Molnár et al. found that children’s mean anxiety levels were 8.45% lower for rabbit-present intervals compared to rabbit-free intervals (generalized linear model) (22). This change corresponded to a change from a slightly stressed anxiety level to normal. Stratifying by school type, the authors identify that the effect was more pronounced in integrating schools (i.e., those with higher percentage of children with poorer socio-economical background, behavioral disorders, and special education needs (SEN); −9.48%) than in non-integrating schools (−7.24%).

Iváncsik et al. found that both, state- and trait anxiety were significantly lower during the rabbit-assisted periods and that the change in mean anxiety was about nine magnitudes larger for children with high baseline trait-anxiety (mean score: 5.41), than for non-anxious children (mean score: 0.60) (23). When zoning in on specific six-week intervals, decreased state anxiety was significant during the 6th and 12th week (albeit noticeable but not statistically significant in other periods as well). Of note, these weeks corresponded to the first time a rabbit was introduced to class. Both studies are based on small sample sizes (n = 27 and n = 16, respectively).

The study by Meints et al. is an RCT resulting from a larger research initiative in the UK (LEAD program: Lincoln Education Assistance with Dogs) (24, 25). The authors examined the effect of HAI with dogs on salivary cortisol as a proxy for stress in primary school children with and without special education needs. A total of 90 (from 105 initially) children from mainstream schools and 44 children with SEN participated. The study period included eight sessions scheduled over 4 weeks (plus 1 week pre- and post-intervention). Children were randomly assigned to either spend ~20 min learning about and interacting with a dog (monitored by a handler), have a guided relaxation session, or have class as usual. The interventions were further divided into individual sessions and group sessions with up to seven children. Salivary cortisol was measured at the beginning and end of the program. Before and after three of the relaxation/dog interventions, a subset of 47 children from the mainstream schools provided additional salivary cortisol samples to measure the “acute” effects of the sessions. Baseline cortisol did not differ between children with or without a dog at home. Comparing school types, those without special education needs presented with greater differences in cortisol levels between pre- and post-intervention than the children with SEN. At the end of the study period (also end of term) cortisol levels in children with and without SEN were similar. Looking specifically at children in the mainstream school, cortisol levels increased from the pre- to post- periods in the ‘no intervention’-group (*p* = 0.017) and in the relaxation group (*p* = 0.025) but not in the HAI group (*p* = 0.212). Similar effects were observed when stratifying by individual and group interventions. In the acute setting, cortisol levels were lower both after the relaxation and the HAI interventions compared to pre-intervention levels. Looking specifically at children with SEN, there were no significant pre vs. post differences in cortisol levels for any study groups (the HAI group was the only one which presented with – non-significantly – lower mean cortisol post compared to pre).

Overall, the studies from the pre-university sector indicate a possible benefit, but there are only few and small studies.

The empirical studies were not evaluated meta-analytically due to their high degree of heterogeneity (results, session characteristics, species, timing).

## 4 Discussion

Clearly, the use of HAI is increasing and benefits against stress in academic settings appear possible. Media reports from Oxbridge highlight the popularity of HAI. Studies from the university setting indicate benefits; however, it is unclear what the optimal duration and frequency of HAI may be. Studies from the pre-university setting also suggest benefits; however, the studies are few in number and low in sample size. In either setting, suggestions of short-term effects cannot be generalized to long-term benefits of HAI in coping with academic stress.

Oxbridge has seen an initiative to push the HAI agenda in 2017: as an April Fool’s joke the Oxford University’s Student Newspaper reported that the university would be the first higher education institution in the UK to offer its students temporary care for a canine friend to improve student wellbeing (26). The authors hoped that this should prompt the university to introduce similar programs in the future.

There is particular scope for further research at the pre-university level as only three studies fit our eligibility criteria. Limited by a small sample size and low methodological quality, Molnár et al. report that school type and sex had a stronger influence on anxiety scores than the presence of the rab-bit (22). However, since the presence of a rabbit is a modifiable factor, it offers practical value as an intervention, especially when children are first introduced into primary school. Since Iváncsik et al. only report a significant reduction in anxiety during the first rabbit intervention, confounding and/or training effects/diminishing returns might play a role (23). This intervention-interval is temporally closest to the school start of term and therefore the association may be confounded by other adaptive mechanisms. Habituation to the HAI may diminish the impact of interactions. This may help ex-plain why, after a certain settling-in period, the effect of the rabbit HAI is no longer significant. Regarding differences between neurotypical and SEN children, Meints et al. found that children benefit differentially from HAI (24). While neurotypical children benefitted from both individual and group sessions, SEN children only benefitted from the HAI in a group setting (cortisol levels increased in the individual sessions and were higher for all SEN children to begin with). This suggests that the positive effects may be attributed to the dynamics of the group context rather than the presence of the dog per se. Possible contributing factors included peer support, increased opportunities for social interaction, reduced individual pressure, and the dog’s role as a social catalyst – elements that are particularly important for SEN children who often struggle with social, emotional, and behavioral challenges. Furthermore, the effect of HAI on people in academic settings can be influenced by individuals’ personal attitudes and beliefs toward animals. People who perceive animals positively may benefit more from HAI, for example through stress reduction, increased engagement, and improved learning outcomes (5). On the other hand, people who fear or dislike certain animals are expected to benefit less from the advantages of HAI. Therefore, personal beliefs or emotional responses toward animals – such as fear of dogs – can act as mediating factors that influence the effectiveness of stress reduction and other positive outcomes associated with HAI.

Regarding details of our literature searches, screening additional databases such as PsycINFO or ERIC may have offered further insight into the topic. However, since we worked with two of the largest life-science databases (PubMed and Web of Science), we expect appropriate coverage.

Overall, the limited evidence from pre-university settings is in line with the findings from university settings and also with a systematic review on support animals for healthcare staff (7). In the latter, HAI was generally well received, with reported benefits including reductions in feelings of stress and anxiety among healthcare professionals.

### 4.1 Requirements for animals in academic settings

There are several hurdles before introducing animals into any public setting – be it a university, school, or hospital. These include concerns about hygiene, fear of animals, and the risk of allergic reactions (e.g., asthma). In primary schools, no child had to be excluded from the study by Iváncsik et al. due to any of the aforementioned (23). Nevertheless, preventive measures should always be considered. While continuous fecal monitoring for pathogens such as Salmonella or Campylobacter – as conducted by Molnár et al. and Iváncsik et al. – may be impractical in everyday settings, several pre-cautions can help minimize health risks in school-based animal interactions (22, 23). Animals selected for HAI should undergo veterinary examination, be vaccinated, treated for parasites, and free of zoonotic diseases. Where applicable, animals should have their claws trimmed and be properly trained for their specific roles to maximize safety. On the other hand, in view of the “hygiene hypothesis” or the “microbial deprivation hypothesis,” introducing animal contact to children could potentially help prevent development of atopic disorders (27).

When planning HAI, and regardless of academic setting (pre-university, university), animal welfare and ethics are also key. Herein, all of the included studies may act as a blueprint. Molnár et al. and Iváncsik et al. pre-selected rabbits that were bred to exhibit key traits suited to the classroom setting (22, 23). This included early habituation to human interaction and the selection of particularly bold individuals with low baseline cortisol levels. To avoid the animals being stressed during the interventions, the studies adhered to the Guidelines for animal welfare in classroom settings by the Royal Society for the Prevention of Cruelty to Animals (RSPCA). Similarly, Meints et al. followed a welfare protocol for dogs, including risk assessment, environmental familiarization, and clear behavioral guidelines (24). Furthermore, animals require daily care, including feeding and cleaning their enclosures. In the examples discussed above, these tasks were carried out by volunteer children who fed the rabbits twice a day and maintained the cages by removing soiled litter. However, the specific context of the school must be considered. In special needs schools, for instance, integrating such routines may not be as straightforward. Ultimately, a teacher will be responsible, either to take on the care themselves or to assist students in doing so. This additional duty could offset HAI benefits on students by having the teacher experience increased stress – especially at a time when educators are already facing significant workloads and are at high risk for burnout (28, 29).

### 4.2 Further perspectives

Some students may wish to take on the additional responsibility of an extra companion, regardless of the restrictions of University regulations. The death of a ‘pet’ snake kept by a student shows that this can go very wrong (30).

Moreover, there is potential value of interaction with pets for creative work. Polly Matzinger, a renowned immunologist, repeatedly emphasized that walks with her dog Annie contributed to her scientific work (31). The physicist Jack H. Hetherington even went so far as to list his cat, Chester, as a co-author on one of his papers, under the name F. D. C. Willard (31).

## 5 Conclusion

HAI may be beneficial in academic settings but more research is required, in particular for pre-university settings.

The concept of “brief, frequent contact” explored here may serve as a starting point for future research into effects of HAI on academic stress (e.g., standardized outcomes, simultaneous measurement of stress in students and animals, cluster RCTs).

To close with Byron, the Lord had more ideas to affect academic stress when he asked for his furry companion to be enrolled as a student. The university’s “no” prevented the bear from sitting next to the students in lectures! In this historical case, the departure of the “pet” with the Lord in 1808 must have meant less stress on Cambridge as a whole.

## Data Availability

The original contributions presented in the study are included in the article/supplementary material, further inquiries can be directed to the corresponding author.
